# Supplementary material for: Chromothripsis during telomere crisis is independent of NHEJ, and consistent with a replicative origin
Source: Genome Res. 2019 May;29(5):737–49. doi: 10.1101/gr.240705.118 (PMC6499312; doi:10.1101/gr.240705.118)
Supplement: Supplemental Material [file supp_gr.240705.118_Supplemental_file_1.zip › contigs/annotated_contigs/DB79/contig.2.DB79_length_714_mean_cov_13.4397759104.docx]

**DB79_length_714_mean_cov_13.4397759104**

```
TACAATTCAGGAGAAGCCATTCAGGAAGAGATGCACAGGGCAAGGTGGGCAGAAGGGAAAGGGGTATAGAGCTTCCACATCATCTCTGG
 >chr9:115761967-115762355 + E=1e-218
GCATGCGACACTTCCAGCACCTTGATGTGTAGACCAATCTAGAAGGTCATCAAAGATCACTGTTTAAGAATTTGTATAGAGCTTAATCT

CCATCCCCATACCTTCCTTCCCAGAGGTTAGTTGGGTGGAGTTGAAAGTTATAACCCTCTAGTTGCTTGGTCTTTTTAGTGATCAGATC

CACTATTGAGGCTATCTAGGGGCCCTACCCTATTTCACCTCATCCACATAAACTCAAGTATGATTTTTTAAAAAGGTGTATTATGAACA

ACAAAAGATATTCCTATCACTCAGGAAAT|CTG|ATTCTTGGGCCCTATCCCAGACTTTTTTATTCAATAGGTCTGGTGTTGGGCCTAG
                              >chr9:123403866-123404195 + E=4e-186
GAATTTGCATTTCTAAGCAATTTCCAGGTTAAACTGATATTGCTGGTCTGGGGACCATAATTAGAGAACTACTCTACTAAATTAACTGT

ACTTATTTCACTCTATTTTTTATATCCCTATCCCTGACTGGTAAAGCTACTTACTTTCCACTTCCAGGCATAGTGATGAACCCTGAATT

TGGTATCAATTCTAACCTAAGAAGAGGGTACTTAAGGCAATCAACCTCAGCGGTCTGTCAAGTCCCATTTGCTACTGGTTCCTTTGTCC

TCAG
```
